# Effectiveness of Dry Needling and Ischemic Trigger Point Compression in the Gluteus Medius in Patients with Non-Specific Low Back Pain: A Randomized Short-Term Clinical Trial

**DOI:** 10.3390/ijerph191912468

**Published:** 2022-09-30

**Authors:** Sara Delgado Álvarez, Jorge Velázquez Saornil, Zacarías Sánchez Milá, Gonzalo Jaén Crespo, Angélica Campón Chekroun, José Manuel Barragán Casas, Raúl Frutos Llanes, David Rodríguez Sanz

**Affiliations:** 1 FisioSalud Ávila, 05002 Ávila, Spain; 2 Department of Physiotherapy, Universidad Católica de Ávila, 05005 Ávila, Spain; 3 Department of Physiotherapy, Universidad Europea de Madrid, 28670 Madrid, Spain; 4 Campus San Jerónimo Guadalupe, Universidad Católica de Murcia, 30107 Murcia, Spain; 5 Facultad de Enfermería, Fisioterapia y Podología, Universidad Complutense de Madrid, 28040 Madrid, Spain

**Keywords:** low back pain, myofascial pain syndrome, trigger points, physiotherapy techniques, manual therapies

## Abstract

Background: The presence of latent myofascial trigger points (MTrPs) in the gluteus medius is one of the possible causes of non-specific low back pain. Dry needling (DN) and ischemic compression (IC) techniques may be useful for the treatment of these MTrPs. Methods: For this study, 80 participants were randomly divided into two groups: the dry needling group, who received a single session of DN to the gluteus medius muscle plus hyperalgesia (*n* = 40), and the IC group, who received a single session of IC to the gluteus medius muscle plus hyperalgesia (*n* = 40). Pain intensity, the pressure pain threshold (PPT), range of motion (ROM), and quality of life were assessed at baseline, immediately after treatment, after 48 h, and one week after treatment. Results: Statistically significant differences were shown between the two groups immediately after the intervention, showing a decrease in PPT (*p* < 0.05) in the DN group and an increase in PPT in the IC group. These values increased more and were better maintained at 48 h and after one week of treatment in the DN group than in the IC group. Quality of life improved in both groups, with greater improvement in the DN group than in the IC group. Conclusions: IC could be more advisable than DN with respect to UDP and pain intensity in the most hyperalgesic latent MTrPs of the gluteus medius muscle in subjects with non-specific low back pain, immediately after treatment. DN may be more effective than IC in terms of PPT, pain intensity, and quality of life in treating latent plus hyperalgesic gluteus medius muscle MTrPs in subjects with non-specific low back pain after 48 h and after one week of treatment.

## 1. Introduction

Low back pain (LBP) is defined as pain located below the last ribs and above the buttocks, with or without pain in the lower extremities [1,2,3,4]. LBP is a major public health problem worldwide; it is estimated that 80% of the adult population will have difficulty with low back pain in their lifetime [5,6,7,8,9,10]. Studies indicate that the prevalence of non-specific low back pain worldwide is at 84% [1], increasing with age between 60 and 65 years, and gradually decreasing thereafter [2]. Between 1990 and 2015, a 54% increase in disability caused by low back pain was observed in low- and middle-income countries, making it one of the leading causes of disability worldwide [11,12].

The prevalence of non-specific LBP is higher in women than in men; some authors believe that this is due to anatomical and functional issues. Women have lower muscle mass, shorter stature, lower bone density, and greater joint fragility [9]. Most people who experience low back pain have recurrent episodes. The annual incidence rate ranges from 15% to 45%, being higher in the third decade [2,3].

There are risk factors that may favor the appearance of non-specific LBP, such as certain habitual or professional postures that cause spinal deviations, excess weight, and the distension of the abdominal wall [9]. Most people do not identify the pathological cause of their low back pain; only a small percentage of the population knows the cause, with 90% of cases being non-specific [9,10]. Therefore, non-specific LBP is characterized by its not having an identified medical origin [1]. R.D. Gerwin reported that 85% of back pain is associated with myofascial pain syndrome [13].

### Myofascial Pain Syndrome (MPS) and Myofascial Trigger Points (MTrPs)

Myofascial pain syndrome is a regional pain syndrome that is characterized by a set of sensory, motor, and autonomic symptoms caused by the presence of myofascial trigger points located in a tight band of skeletal muscle, tendons, or fascia, which are painful upon compression and may be accompanied by local or referred pain [3,5].

Trigger points are considered to be the most sensitive point of a contracted muscle band that can be felt on palpation. One hypothesis postulates that this shortening is due to an excessive release of acetylcholine by the motor endplates [5]. According to their clinical characteristics, myofascial trigger points can be classified as active or latent. Active MTrPs are sensitive and identifiable by the patient with compression, causing pain, restriction of movement, and weakness at rest. One of their most important characteristics is referred pain, which is similar to the patient’s pain complaint and is located far away from the site of origin of that point [14]. When the trigger point is adequately stimulated, a local contraction response in the muscle fibers occurs, producing referred and autonomic motor phenomena. As they are modulated by the sympathetic nervous system, they can be conditioned by states of anxiety and stress [5]. On the other hand, latent MTrPs do not cause spontaneous pain; they are inactive and are only painful when palpated. They have the same clinical characteristics as active ones but can also limit movement and cause muscle weakness [5,14]. Latent MTrPs may develop after sustained muscle contractions over long periods of time or following repeated physical activity. The pain of latent MTrPs can be activated by digital compression, stretching, or overloading [15]. At least three of the following diagnostic criteria must be met for MTrPs to be present [15,16,17]:

- The presence of a palpable and tense band.
- Local and pressure-referred pain in the nodule of the tense band.
- The patient’s recognition of pain.
- Limited range of motion.

The most common symptoms are persistent local or referred pain, accompanied by a decreased range of motion, as well as motor dysfunction of the affected muscle, fatigue, and muscle weakness [18,19,20,21,22,23,24]. The pain is reproducible and does not follow a dermatomal or nerve root distribution. Active MTrPs always present spontaneous pain, while latent MTrPs only present pain during mechanical stimulation [25]. Studies have determined that the presence of MTrPs is the first sign of muscle overload; they not only affect the local muscle level but also produce sensitization of the central and peripheral nervous system, interaction with the visceral somatic system, alteration of the microcirculation, and proliferation of inflammatory mediators [17,26].

Two of the methods that have the most evidence for the treatment of MTrPs are IC and DN [27,28,29,30,31,32,33,34,35,36,37]. Studies show that DN is considered one of the most effective techniques for the direct inactivation of MTrPs, improving symptomatology and relieving pain [6,8]. It is an invasive technique, one that should be performed by a qualified healthcare professional, in which a thin, filiform needle, similar to those used in acupuncture, is inserted into the skin and underlying tissues, causing a change in body function and structures, for the management of pain, range of motion, and disability in musculoskeletal disorders [3]. There are several techniques in the field of deep DN:

- Gunn’s intramuscular stimulation technique: this was one of the first techniques used in the dry needling approach. Gunn considered myofascial pain to be secondary to neuropathy. The technique consists of puncturing the deep paravertebral muscles of the segments related to the patient’s painful areas and the shortened superficial muscles [38,39,40].
- Hong’s rapid entry–exit technique: this technique is the most commonly used and consists of puncturing the MTrPs by rapid entry and exit. It has been shown that the local twitch response (REL), which is the sudden and involuntary contraction of a set of muscle fibers, can interrupt the noise of the motor end plate, causing an analgesic effect [34]. Therefore, studies have shown that DN is more effective when REL occurs, due to the rapid depolarization of the muscle fibers involved, leading to greater pain relief and improved range of motion [41].
- Ischemic compression (IC) is considered one of the most commonly and effectively used non-invasive techniques for the treatment of MPS [7]. It consists of a manual therapy technique in which pressure is applied for 90 s with the thumb part of the hand on the MTrPs, starting with light pressure until the patient’s discomfort sensation or tissue barrier is reached, maintained until the sensation of discomfort or the tissue barrier disappears, and thereafter increased and progressed according to the patient’s tolerance [30].

Both DN and IC are techniques that aim to deactivate the MTrPs. Strong evidence has shown the immediate relief of symptoms produced by MTrPs [7,42,43,44,45,46,47,48]. The effects of IC include the normalization of muscle fiber biomechanical properties, restoring muscle function by improving the osteoarticular range of motion, stimulating mechanoreceptors, and decreasing pain signals due to the depletion of neurotransmitters and the temporary blockage of blood flow [49,50,51].

The aim is to test the efficacy of these two possible treatments for non-specific low back pain in the short term by measuring different variables.

## 2. Materials and Methods

### 2.1. Design

A single-blind, randomized, two-group clinical trial was conducted, in which the assessor was blinded to evaluate the short- and medium-term effectiveness of single-session DN treatment versus single-session IC treatment for latent MTrPs in the gluteus medius muscle, in patients with non-specific low back pain. Four measurements were performed: quality of life, range of motion, pain intensity, and PPT. The quality-of-life questionnaire was performed pre-intervention and at the end of treatment. The rest of the measurements were performed pre-intervention, immediately after treatment, and 48 h and one week after the treatment session. Before participating in the study, all subjects read and signed the informed consent form, which consisted of a description of the pathology, the procedures to be performed, and their risks and benefits.

During the first session, after they signed informed consent forms, all participants were randomized to ensure adequate blinding. Each participant received the assigned intervention (i.e., DN or CI). All participants were randomized to each other as a control for the sequential effect of order and transfer; this was conducted by an independent investigator using Epidat 3.1 (www.sergas.es (accessed on 1 April 2022)). Participants were blinded to the assigned intervention; in addition, all participants were blinded to the use of the DN procedure. The researchers were also blinded, in that the researcher involved in data acquisition was different from the researcher delivering the intervention.

### 2.2. Participants, Therapists, and Centers

Before participating in the study, all subjects read and signed the informed consent form, which consisted of a description of the pathology, the procedures to be performed, and their risks and benefits. Compliance with the Declaration of Helsinki, the Biomedical Law, the Law on Patient Autonomy in Data Processing, and Organic Law 03/2018 of 5 December on the Protection of Personal Data and the Guarantee of Digital Rights was recorded. In addition, the study was approved by the research committee of the Complejo Asistencial de Ávila, code GASAV/2022/08, and has been registered with clinicaltrials.gov as NCT05440253.

The participants in this study were patients chosen from the physiotherapy clinic of Fisiosalud Ávila from February to May 2022; these patients were previously diagnosed by an orthopedic surgeon by means of a complete set of orthopedic and complementary diagnostic tests, after which the clinical judgment was of non-specific low back pain. After that, a clinical examination was carried out by the principal investigator to check for the presence of latent MTrPs in the gluteus medius and identify the selection criteria in the selected participants. For palpation of the taut band of the MTrPs of the gluteus medius, an inter-examiner and intra-examiner reliability study was carried out to avoid biases in the intervention.

INCLUSION CRITERIA

- Signed informed consent form.
- Presence of non-specific low back pain for more than six weeks.
- Presence of a palpable tight-band nodule in the gluteus medius muscle.
- Presence of a hypersensitive or hyperirritable point in the tension band.
- Patients reporting local or referred pain in the area of the latent MTrPs after mechanical stimulation.

EXCLUSION CRITERIA

- Surgeries in the lumbopelvic region.
- Diagnosed herniated discs in the lumbar region.
- Patients with neurological alterations.
- Lower limb length discrepancy (>0.5 cm)
- Age outside the range of 18 to 75 years.
- Ingested or injected anticoagulant or antiplatelet drugs.
- Systemic or local infection in the lumbar region.
- Pregnancy.
- Presence of fear of needles (belonephobia).

### 2.3. Intervention

A single-blind clinical trial was conducted using a two-group randomized design, where the assessor was blinded to evaluate the short- and medium-term effectiveness of single-session DN treatment versus single-session IC treatment for latent MTrPs in the gluteus medius muscle, in patients with non-specific low back pain.

#### 2.3.1. Common Parts of Treatment

The participants included in the study received an intervention that included 4 assessments: pre-intervention, post-intervention, 24 h after treatment, and one week after the intervention. Initially, both groups underwent the identification of latent MTrPs in the gluteus medius muscle. Both groups were assessed with four measurements: quality of life, pain intensity, pain threshold upon pressure, and quality of life.

#### 2.3.2. Identification of Latent Trigger Points

Latent MTrPs were detected by the same physiotherapist who performed the pre-intervention and post-intervention measurements while blinded to the treatment assignment. The presence of latent MTrPs was confirmed by the researcher who carried out both interventions. The most hyperalgesic latent MTrPs of the gluteus medius was selected and marked with a cross using a permanent marker, to be assessed and treated [52,53,54,55,56]. These latent MTrPs were defined as the most hyperalgesic or hyperirritable nodule in a tight band that is activated or that generates local or referred pain when palpated by digital compression and that generates limitation in the range of motion upon stretching [15]. In the case of the gluteus medius, this point is usually located close to the iliac crest in the posterior part of the muscle, next to the sacroiliac joint [57].

#### 2.3.3. Invasive Procedure: Dry Needling Group (*n* = 40)

First, the physiotherapist wore sterile gloves and cleaned the area to be treated with alcohol, taking the necessary precautions. The patient was arranged in a prone position, after locating the most hyperalgesic latent MTrPs of the gluteus medius muscle within the taut band, a stainless-steel needle of 0.25 × 60 mm in length (Agupunt, Madrid, Spain) was introduced, held in the therapist’s dominant hand, gripped firmly between first and third finger and introduced perpendicularly to the ileum. The needle made contact with the ileum and was used as a reference to ensure that the depth of the needle was adequate to reach the muscle. The thumb was placed on the anterior part of the MTrPs and the second and third finger of the therapist on the posterior part of the MTrPs below. The needle was moved up and down, using Hong’s [34] “fast in and fast out” technique, with a frequency of approximately 1 to 2 strokes per second without removing the needle completely from the skin. The technique was applied up to the patient’s tolerance limit or up to a maximum of 8–10 insertions, in order to obtain the maximum amount of REL [41,58,59,60,61,62,63,64,65,66,67,68,69]. Upon completion of the technique, the needle insertion site was firmly compressed with the thumb for three seconds and the needle was then discarded in a sharps needle container.

#### 2.3.4. Conservative Procedure: IC Group (*n* = 40)

Patients were placed in a prone position. After locating the most hyperalgesic latent MTrPs of the gluteus medius muscle, the physiotherapist applied pressure with the thumb of his dominant hand on the selected MTrPs until the patient’s pain threshold changed from a pressure sensation to pain. This technique was maintained for 90 s and repeated up to three times [30].

### 2.4. Outcome Measures

Primary outcome: pain intensity

The visual analog scale (VAS), one of the most commonly used methods in the literature for the assessment of pain, was used to measure pain intensity. It consists of a horizontal line of 10 cm; on the far left is the number 0, representing the absence of pain or minimum pain intensity, and on the far right is the number 10, representing maximum pain intensity [70]. This measurement process was performed with all subjects, at the time of pre-treatment, immediately post-treatment, 48 h post-treatment, and one week after treatment. Patients were instructed to mark their perceived pain level using this scale; once they had made their mark, the distance between the initial end and their mark was measured. The data obtained were recorded in a pain record table, together with the data obtained from the PPT.

Secondary outcome: quality of life

This measurement was carried out using the Oswestry disability index, which, together with the Roland–Morris scale, is the most commonly recommended instrument and is used worldwide as a scale to measure disability related to low back pain [68]. Together with other complementary tests, this type of assessment scale provides us with substantial information on the intensity of the patient’s pain, as well as its repercussions in their daily life and activities, as studies have shown that low back pain is the main cause of absence from work and for referrals for medical rehabilitation in recent years [12,68].

This scale has been translated into Spanish and has demonstrated its reliability, validity, and internal consistency, being included in the category of “highest methodological quality” with recommendation A (a high level of development) [69].

The scale consists of 10 questions, with 6 possible answers to each one, that are easy to understand; each item is rated from 0 to 5, from the least to the most limited. If an item is not answered, it is excluded from the final calculation. The total score is expressed as a percentage, with a maximum of 100%, and is obtained by adding the results of each item, dividing that by the maximum possible score, and multiplying it by 100 [68].

The scale considers 0–20% as minimal limitation or disability, 21–40% as a moderate limitation or disability, 41–60% as a severe limitation or disability, 61–80% as disabled, and over 81% as a maximum offunctional limitation [68].

This scale was carried out on all subjects in the time prior to the intervention and one week after the intervention, when it was briefly explained to them; they then filled in the questionnaire on their own, without the presence of the researcher, to avoid the possible intimidating effect of the staff.

- PRESSURE PAIN THRESHOLD

The PPT can be defined as the minimum pressure intensity or stimulus at which the patient perceives pain [71]. This measurement was assessed in the previously selected latent MTrPs using the Wagner FORCE DIAL FDK 60 analog algometer (Wagner Instruments, Greenwich, CT, USA). This tool showed high reliability for assessing the effect of treatment in patients with MPS [72]. It was applied perpendicular to the latent MTrPs of each subject, increasing the pressure with a progression of 1 kg per second. The measurement was stopped when the patient reported pain. Prior to the measurement, patients were trained and familiarized with the algometer, in order to be made aware of the sensation and to be able to correctly indicate when the sensation changed from pressure to pain. A total of four measurements were taken, one pre-intervention, one immediately after, one after 48 h, and one after one week.

- RANGE OF MOTION (ROM)

Studies have shown that both DN and CI are effective techniques for immediate pain relief, as well as for improving ROM in patients with MPS [53]. The Schober test, which measures the degree of flexibility of the lumbar spine in the flexion movement, has moderate validity and offers very good reliability in patients with low back pain [72,73]. The patient was initially placed in a standing position; a mark was made with a permanent marker pen on the skin in the area corresponding to the spinous process of the S1 vertebra, then, a tape measure was used to measure 10 cm cranially along the spine and another mark was made. Firstly, the patient was asked to make an anterior flexion, as if he/she wanted to touch his/her toes, and the distance between both marks was measured; this should increase by 5 cm. Secondly, the patient was asked to make a lumbar extension, and the distance between both marks was measured again. The initial measurement should be shortened by 1 to 2 cm; if these measurements are not reached, there is a limitation of the lumbar spine [73].

### 2.5. Data Analysis

To carry out this statistical analysis, the IBM SPSS statistical software (SPSS 24 Inc., Chicago, IL, USA) was used. First, the descriptive statistics of the control or independent variables were carried out to check whether there was a normal distribution, then the Shapiro-Wilk test was performed. Similarly, a descriptive statistical analysis of the dependent variables of the study was performed to check for normal distribution. 

Subsequently, the homogeneity of the sample was checked. For quantitative variables that followed a normal distribution (*p* > 0.05), the parametric Student’s *t*-test for independent samples was used. For those quantitative variables that did not follow a normal distribution (*p* < 0.05), the non-parametric Mann–Whitney U-test was used. To assess the homogeneity of the sample in terms of nominal variables at the pre-intervention point, the chi-squared test was used.

To analyze the different measurements taken at pre-intervention, post-intervention, after 48 h, and one week after treatment in each group, a repeated measures analysis was performed. For variables following a normal distribution, a general linear repeated measures model with two factors was used. The within-subject factor was defined as the time of data collection, consisting of four levels: pre-intervention, post-intervention, after 48 h, and after 1 week. The second factor, inter-subject, was formed by the group variable, consisting of two levels: the DN group and the IC group.

To compare the differences that existed between the different moments of the measurements between groups, a post hoc analysis was carried out using the Bonferroni method. The Mauchly test (*p* < 0.05) was used to check that the variables met the assumption of sphericity. For those variables that did not meet Mauchly’s sphericity assumption (*p* > 0.05), the Greenhouse–Geisser correction was used.

## 3. Results

**Figure 1:**
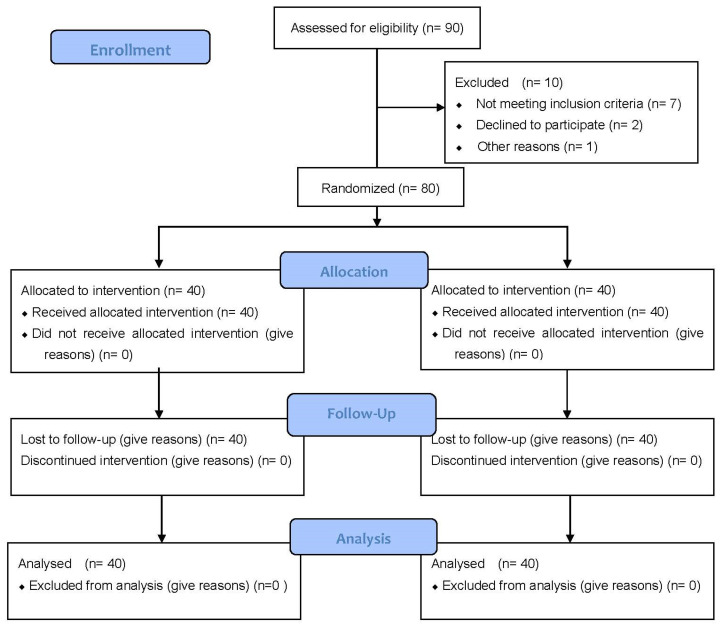
Flow Diagram.

Out of a total of 90 individuals recruited, 10 subjects were excluded from the study: *n* = 7 subjects because there were no MTrPs at screening, *n* = 2 subjects for not appearing for the screening, and *n* = 1 subject excluded because of pregnancy. The subjects did not present any adverse effects, nor were there any losses due to participants’ absence from the assessment or because of the use of analgesic medication (Figure 1).

Table 1 shows the descriptive statistics of the control and independent variables. 

There were no statistically significant differences between subjects in the DN group and the IC group on these control variables (Table 2).

Table 3 shows the descriptive statistics of the dependent variables recorded in the study.

Significant differences were found (*p* < 0.05), calculated by the linear method of repeated measures, using post hoc analysis with the Bonferroni method, whereby significant differences were found between the groups and the time of sampling in the dependent variables via VAS, algometry, and the Oswestry index. No statistically significant differences were found in the Schober test between the groups and at the time of intake, where the value was *p* = 0.167 in both groups after one week of the intervention.

PAIN INTENSITY: VAS

Table 4 shows the descriptive statistics for the measurement of pain intensity. It was found that the distribution of this variable was not normal; therefore, comparisons were carried out using non-parametric tests. At the time prior to the intervention between the two groups, the Mann–Whitney U-test showed that pain intensity was greater in the IC group than in the dry needling group. Post hoc analysis after treatment showed statistically significant differences (*p* < 0.05) between the groups.

ALGOMETRY

Descriptive statistics for the pain threshold regarding pressure in the gluteus medius muscle can be found in Table 5. No statistically significant differences (*p* > 0.05) were shown in the linear repeated measures analysis at the pre-intervention time between the two groups.

RANGE OF MOTION: SCHOBER TEST

Table 6 shows the descriptive statistics of the Schober range-of-motion test. When comparing the measurements between the two groups at the pre-intervention time, using the Chi-square test, no statistically significant differences (*p* > 0.05) were found between the two groups. In addition, no statistically significant differences (*p* < 0.05) were found between the pre- and post-intervention time (at one week) between the two groups in terms of range of motion.

OSWESTRY QUALITY OF LIFE QUESTIONNAIRE

Table 7 shows the descriptive statistics of the Oswestry quality of life questionnaire. In the exploratory analysis of this variable, no statistically significant differences were found at the pre-intervention time between the groups (*p* > 0.05). Both groups improved their score on the quality of life questionnaire after one week of treatment, with the mean score being lower in the DN group.

## 4. Discussion

Studies have shown that IC and DN are effective techniques for immediate pain relief and the improvement of ROM in patients with upper trapezius trigger points and neck pain [53,54,55]. Both techniques have been shown to be useful in patients with short-term upper quadrant body pain [57]. It has also been shown that both techniques can be a useful adjunct for chronic low back pain [46], with the techniques being more effective in the short term in reducing pain than sham or placebo techniques [65,66,67]. This study can be considered the first clinical trial to determine the effectiveness of DN and IC on latent MTrPs of the gluteus medius in patients with non-specific low back pain, regarding pain intensity, pain threshold to pressure, ROM, and quality of life, both in the short term and one week after treatment. Other studies have shown no beneficial effects on pain intensity and ROM for both techniques [74,75,76]. The results of the present study have shown significant differences in pain intensity, pain threshold to pressure, and quality of life immediately after the intervention and at a 1-week follow-up in both techniques, the results being more favorable regarding DN, compared to IC.

PAIN INTENSITY: VAS

Dry needling is effective in improving pain and functional balance in patients with low back pain [46] and is increasingly used for the treatment of various ailments without the need for other associated treatments, giving good results. This technique has been shown to reduce pain immediately after treatment in patients with musculoskeletal problems [47,48]. However, in our study, the results show that immediately after treatment the intensity of pain worsened, being greater in the DN group, improving after 48 h, and the improvement is maintained and even increased one week after treatment. These results justify and support studies that have shown that pain and hyperalgesia are present in all subjects after DN treatment of latent MTrPs, usually lasting less than 72 h [77,78].

This post-puncture pain is thought to be a consequence of neuromuscular damage and the hemorrhagic and inflammatory reaction generated by the needle, activating the descending inhibitory mechanisms of pain [43], reducing the segmental nociceptive afferents of the trigger point and acting via central sensitization [44,45].

IC has been shown to be a highly effective technique for the treatment of MTrPs, producing immediate pain relief [7]. Other studies show that MTrPs compression in subjects with chronic low back pain can modulate prefrontal cortical activity and may relieve pain [79]. These studies reinforce our results, where there is immediate pain relief in subjects receiving IC, compared to those in the DN group, demonstrating a short-term effect. However, the improvement does not increase over time.

PAIN THRESHOLD RESPONSE TO PRESSURE

Some studies have found no statistically significant change in pain intensity [74,75], while others have shown that dry needling is considered one of the most effective techniques for the direct inactivation of PGMs, improving symptomatology and relieving pain [6,8,51,77,80,81,82,83]. It has been shown that the local spasm response (REL) can interrupt the noise of the motor end plate, causing an immediate and longer-lasting analgesic effect than if RELs are not produced [80]. This is due to the rapid depolarization of the muscle fibers involved, leading to greater pain relief and an improved range of motion [41]. Our study, as with those previously cited, used the “Hong fast-in and fast-out” technique, either up to the limit of the subject’s tolerance or 8–10 insertions, in order to obtain the greatest number of local twitch responses.

Studies have demonstrated a lower pain PPT, immediately after a single session of DN treatment, on myofascial trigger points in the thoracic, lumbar, and trapezius muscles [80,81,82]. Our results agree with these studies in the post-intervention measurement, where a lower pressure pain threshold was observed in the DN group than in the IC group. These scores changed at 48 h and one week after treatment, when there was an increase in the PPT. This is comparable to previous studies, where researchers have shown that PPT levels increase when dry needling is performed, and the PPT level may be higher two days after the intervention [53]. Another study has even found that the increase in the pressure pain threshold continues to increase and is maintained during the two weeks following treatment in two DN sessions [62]. Our study with a single DN session produced significant effects on the pressure pain threshold, increasing after 48 h, and maintaining the effect one week later. We agree with the above-mentioned studies that the more local the spasm responses obtained while respecting the patient’s tolerance, the more powerful the result will be, and the more lasting the effect will be.

A systematic review has shown that IC has moderate to strong evidence for the immediate relief of myofascial trigger points. Llamas et al. [83] demonstrate that the PPT increases immediately after treatment and is maintained at 48 h after treatment in subjects with cervical pathology. However, in our study, an increase in the PPT is observed immediately after the intervention in the IC group in relation to the DN group, but this improvement is not maintained over time at 48 h or at one week after treatment.

In agreement with other studies [24,84], our IC technique was applied for 90 s until the patient’s PPT changed from pressure sensation to pain and was repeated three times. However, another study has found that where they recommend applications for 60 s below the PPT, without reaching the patient’s discomfort sensation, and for 90 s with elevated pressure, reaching the patient’s pain or discomfort threshold, to increase the PPT and force the latent trigger points of the levator scapulae in the short term [85].

SCHOBER TEST

Studies show that IC and DN are effective techniques for immediate pain relief and range-of-motion improvement in patients with upper trapezius trigger points and neck pain [53,54,55]. Theodoros Loizidis et al. [46] found that DN appears to improve functional balance in patients with low back pain. The Schober test, which measures the degree of flexibility of the lumbar spine when in flexion movements, has moderate validity and very good reliability in patients with low back pain [73]. However, in our study, no statistically significant differences were found in the Schober test between the DN and IC groups at pre-intervention and post-intervention. In both groups, changes were obtained at pre- and post-intervention time, but the sample size was not large enough to be statistically significant, which may be a limitation of this study.

Another study has been found that reinforces our results, where there was also no statistically significant difference in range of motion in patients with latent MTrPs in the shortened gastrocnemius, using DN and IC [75].

QUALITY OF LIFE: OSWESTRY QUESTIONNAIRE

Studies suggest that two sessions of DN and IC produce similar results in terms of pain, disability, and range of motion at the cervical level, with more significant results observed in the DN group [83]. Another study in which DN was performed on subjects with MTrPs in the upper trapezius muscle showed improvements in the DASH disability questionnaire [86].

In our study, as it involved low back pain, the Oswestry low back pain disability index was used, which, together with the Roland–Morris scale, is the most commonly recommended measure and is used worldwide to measure disability caused by low back pain [68]. The results of our study showed significant differences in both groups between pre- and post-intervention results, with a greater improvement in quality of life in the DN group compared to IC.

This study has some limitations. Firstly, both groups were treated by the same physiotherapist, which may limit the generalizability of the data. Secondly, we used a convenience sample based on other studies with similar characteristics, which may be too small for judging greater effect and external validity. Thirdly, the results were assessed immediately after treatment, at 48 h, and one week after treatment, so we cannot be sure that these results will be maintained in the long term. This study has been carried out in a short period of time and should be re-evaluated over a medium-term period (over one to three months’ evolution). These limitations should be taken into account in further research.

## 5. Conclusions

Dry needling at the most hyperalgesic latent myofascial trigger points of the gluteus medius muscle, in subjects with non-specific low back pain, improves pain intensity, the pressure pain threshold, and quality of life more effectively in the short and medium term than is the case with ischemic compression.

Ischemic compression improves pain intensity, the pressure pain threshold, and quality of life immediately after the technique. In terms of the range of motion, no statistically significant differences were found between the two groups (dry needling and ischemic compression).

## Figures and Tables

**Table 1:** Mean (M) and standard deviation (SD) of the control variables, by gender and group.

	Men				Women			
	*Dry Needling Group* *n = 11*		*Ischaemic Compression Group* *n = 28*		*Dry Needling Group* *n = 29*		*Ischaemic Compression Group* *n = 12*	
	M	SD	M	SD	M	SD	M	SD
Age	41.33	15.92	51.57	12.44	52.56	11.63	41.50	12.23
Weight (kg)	78.17	11.26	80.71	8.07	60.44	9.92	63.50	7.61
Height (cm)	177.33	3.88	180.29	2.69	164.44	4.09	166.13	2.80
ICM	24.85	3.64	24.78	2.09	22.32	3.49	22.96	2.199

**Table 2:** Student’s *t*-test for independent samples on the control variables.

	*t*-Student	*p* < 0.05
Age	0.377	0.355
Weight (kg)	−0.868	0.196
Height (cm)	−1.116	0.137
Body mass index	−0.431	0.335

**Table 3:** Mean (M) and standard deviation (SD) of the dependent variables, by gender, group, and time of sampling.

	Men				Women			
	*Dry Needling Group* *n = 11*		*Ischemic Compression Group* *n = 28*		*Dry Needling Group* *n = 29*		*Ischemic Compression Group* *n = 12*	
	M	DT	M	DT	M	DT	M	DT
**VAS**								
Pre-treatment	7.50	0.83	7.86	0.90	7.67	0.86	8.25	1.16
Post-treatment	6.50	1.87	3.71	2.10	7.22	2.72	4.00	2.13
48 h	4.67	1.21	4.29	1.25	4.56	0.88	5.25	1.03
1 week after	4.17	1.72	4.14	4.67	4.22	0.97	5.75	1.69
**ALGOMETRY**								
Pre-treatment	5.26	0.68	5.35	1.32	3.99	0.66	3.96	0.60
Post-treatment	4.66	0.60	5.88	1.02	3.55	0.50	4.73	0.37
48 h	5.58	0.48	5.40	1.17	4.24	0.76	4.61	0.75
1 week after	5.56	0.68	5.34	1.30	4.26	0.65	4.35	0.90
**SCHOBER TEST**								
Pre-treatment	0.33	0.51	0.43	0.53	0.11	0.33	0.13	0.11
1 week after	0.00	0.00	0.14	0.37	0.11	0.33	0.00	0.00
**OSWESTRY**								
Pre-treatment	22.17	6.49	23.86	5.84	19.22	5.63	21.88	5.59
1 week after	15.50	6.09	20.86	4.33	14.67	2.64	20.63	1.92

**Table 4:** Mean (M), standard deviation (SD) and *p*-value of VAS by group and time of acquisition.

	Group	Pre-Intervention	Post-Intervention	48 h	1 Week
M	DN	7.60	6.93	4.60	4.20
	IC	8.07	3.87	4.80	5.00
SD	DN	0.82	2.37	0.98	1.26
	IC	1.03	2.06	1.20	1.81
*p*-value (*p* > 0.05)	DN	0.325	1.000	0.001	0.001
	IC	0.271	0.001	0.001	0.002

**Table 5:** Mean (M), standard deviation (SD), and the *p*-value of algometry, by group and time of measurement.

	Group	Pre-Intervention	Post-Intervention	48 h	1 Week
*M*	DN	4.46	4.00	4.780	4.787
	IC	4.61	5.27	4.98	4.81
*SD*	DN	0.93	0.76	0.93	0.92
	IC	1.20	0.93	1.02	1.18
*p*-value (*p* > 0.05)	DN	0.347	0.025	0.043	0.049
	IC	0.685	0.016	0.473	0.798

**Table 6:** Mean (M) and standard deviation (SD), with *p*-value and Schober test result, by group and time of collection.

	Group	Pre-Intervention	1 Week
M	PS	0.20	0.07
	IC	0.27	0.07
SD	PS	0.41	0.25
	IC	0.45	0.25
*p*-value	PS	0.082	0.167
	IC	0.086	0.167
Positive test result	PS	3	1
	IC	4	1
Negative test result	PS	12	14
	IC	11	14

**Table 7:** Mean (M) and standard deviation (SD) of the Oswestry questionnaire, according to group, and the time of completion of the questionnaire.

	Grupo	Pre-Intervention	1 Week
M	DN	20.40	15.00
	IC	22.80	20.73
SD	DN	5.95	4.17
	IC	5.59	4.78

## Data Availability

The data presented in this study are available upon request from the corresponding author. The data are not publicly available due to compliance with data protection regulations.

## References

[1] Balagué F., Mannion A.F., Pellisé F., Cedraschi C. (2012). Non-specific low back pain. Lancet.

[2] Hoy D., Brooks P., Blyth F., Buchbinder R. (2010). The Epidemiology of low back pain. Best Pract. Res. Clin. Rheumatol..

[3] Griswold D., Gargano F., Learman K.E. (2019). A randomized clinical trial comparing non-thrust manipulation with segmental and distal dry needling on pain, disability, and rate of recovery for patients with non-specific low back pain. J. Man. Manip. Ther..

[4] Ramsook R.R., Malanga G.A. (2012). Myofascial low back pain. Curr. Pain. Headache Rep..

[5] Bron C., Dommerholt J.D. (2012). Etiology of Myofascial Trigger Points. Curr. Pain. Headache Rep..

[6] Hong C.Z. (2006). Treatment of myofascial pain syndrome. Curr. Pain. Headache Rep..

[7] Vernon H., Schneider M. (2009). Chiropractic Management of Myofascial Trigger Points and Myofascial Pain Syndrome: A Systematic Review of the Literature. J. Manip. Physiol. Ther..

[8] Tekin L., Akarsu S., Durmuş O., Çakar E., Dinçer Ü., Kiralp M.Z. (2013). The effect of dry needling in the treatment of myofascial pain syndrome: A randomized double-blinded placebo-controlled trial. Clin. Rheumatol..

[9] Lizier D.T., Perez M.V., Sakata R.K. (2012). Exercises for treatment of nonspecific low back pain. Rev. Bras. Anestesiol..

[10] Hemmer C.R. (2021). Evaluation and Treatment of Low Back Pain in Adult Patients. Orthop. Nurs..

[11] Hartvigsen J., Hancock M.J., Kongsted A., Louw Q., Ferreira M.L., Genevay S., Hoy D., Karppinen J., Pransky G., Sieper J. (2018). What low back pain is and why we need to pay attention. Lancet.

[12] Chenot J.F., Greitemann B., Kladny B., Petzke F., Pfingsten M., Schorr S.G. (2017). Non-Specific Low Back Pain. Dtsch. Arztebl. Int..

[13] Dolor de Origen Muscular: Dolor Miofascial y Fibromialgia. https://scielo.isciii.es/scielo.php?script=sci_arttext&pid=S1134-80462007000100006.

[14] Trigger Points: Diagnosis and Management—American Family Physician. https://www.aafp.org/afp/2002/0215/p653.html.

[15] Borg-Stein J., Simons D.G. (2002). Focused review: Myofascial pain. Arch. Phys. Med. Rehabil..

[16] Vázquez Gallego J., Solana Galdámez R. (1998). Liberación Miofascial: Síndrome de Dolor Miofascial y Puntos Gatillo. https://www.casadellibro.com/libro-sindrome-de-dolor-miofascial-y-puntos-gatillo-liberacion-miofasc-ial/9788495052148/628807.

[17] Shah J.P., Thaker N., Heimur J., Aredo J.V., Sikdar S., Gerber L. (2015). Myofascial Trigger Points Then and Now: A Historical and Scientific Perspective. PMR.

[18] Dolor y Disfunción Miofascial: El Manual de Puntos Gatillo—PMC. https://www.ncbi.nlm.nih.gov/pmc/articles/PMC2484855/.

[19] Unalan H., Majlesi J., Aydin F.Y., Palamar D. (2011). Comparison of high-power pain threshold ultrasound therapy with local injection in the treatment of active myofascial trigger points of the upper trapezius muscle. Arch. Phys. Med. Rehabil..

[20] Capogrossi M.C., Houser S.R., Bahinski A., Lakatta E.G. (1987). Synchronous occurrence of spontaneous localized calcium release from the sarcoplasmic reticulum generates action potentials in rat cardiac ventricular myocytes at normal resting membrane potential. Circ. Res..

[21] Simons D.G. (2008). New views of myofascial trigger points: Etiology and diagnosis. Arch. Phys. Med. Rehabil..

[22] Kalichman L., Vulfsons S. (2010). Dry needling in the management of musculoskeletal pain. J. Am. Board. Fam. Med..

[23] Cummings M., Baldry P. (2007). Regional myofascial pain: Diagnosis and management. Best Pract. Res. Clin. Rheumatol..

[24] Simons D.G. (2004). Travell and Simons’ Myofascial pain and dysfunction: The trigger point manual. Travel Simons’ Myofascial Pain Dysfunct Trigger Point Man.

[25] Bennett R. (2007). Myofascial pain syndromes and their evaluation. Best Pract. Res. Clin. Rheumatol..

[26] Shah J.P., Danoff J.V., Desai M.J., Parikh S., Nakamura L.Y., Phillips T.M., Gerber L. (2008). Biochemicals associated with pain and inflammation are elevated in sites near to and remote from active myofascial trigger points. Arch. Phys. Med. Rehabil..

[27] Fischer A.A. (1987). Pressure algometry over normal muscles. Standard values, validity and reproducibility of pressure threshold. Pain.

[28] Colhado O.C., Boeing M., Ortega L.B. (2009). Toxina botulínica en el tratamiento del dolor. Rev. Bras. Anestesiol..

[29] Hong C.Z., Simons D.G. (1998). Pathophysiologic and electrophysiologic mechanisms of myofascial trigger points. Arch. Phys. Med. Rehabil..

[30] DOLOR Y DISFUNCIÓN MIOFASCIAL (3a ED.): EL MANUAL DE LOS PUNTOS GATILLO|JOSEPH DONNELLY|Casa del Libro. https://www.casadellibro.com/libro-dolor-y-disfuncion-miofascial-3-ed-el-manual-de-los-puntos-gatillo/9788417602024/9857700?gclid=Cj0KCQjw3IqSBhCoARIsAMBkTb0KrnXESbxQL03FjyaPSFzUMzmgGORT6FlUbxFvtPZm2yoiy8GhKlYaAn80EALw_wcB.

[31] Soler-Pérez M.A., del Carmen Serrano-Córcoles M., Ferrer-Márquez M., del Mar López-González M., Pérez-Sáez M.Á., García-Torrecillas J.M. (2021). Evaluación del tratamiento con infiltraciones intraarticulares en la patología osteoarticular del hombro en atención primaria. Aten. Primaria.

[32] Cummings T.M., White A.R. (2001). Needling therapies in the management of myofascial trigger point pain: A systematic review. Arch. Phys. Med. Rehabil..

[33] Lewit K. (1979). The needle effect in the relief of myofascial pain. Pain.

[34] Furlan A.D., van Tulder M., Cherkin D., Tsukayama H., Lao L., Koes B., Berman B. (2005). Acupuncture and dry-needling for low back pain: An updated systematic review within the framework of the cochrane collaboration. Spine.

[35] Langevin H.M., Konofagou E.E., Badger G.J., Churchill D.L., Fox J.R., Ophir J., Garra B.S. (2004). Tissue displacements during acupuncture using ultrasound elastography techniques. Ultrasound. Med. Biol..

[36] Harden R.N., Bruehl S.P., Gass S., Niemiec C., Barbick B. (2000). Signs and symptoms of the myofascial pain syndrome: A national survey of pain management providers. Clin. J. Pain..

[37] Yoon S.H., Rah U.W., Sheen S.S., Cho K.H. (2009). Comparison of 3 needle sizes for trigger point injection in myofascial pain syndrome of upper- and middle-trapezius muscle: A randomized controlled trial. Arch. Phys. Med. Rehabil..

[38] Revisión: Técnica de Punción Seca y Puntos Gatillos Miofasciales. https://www.efisioterapia.net/articulos/revision-tecnica-puncion-seca-y-puntos-gatillos-miofasciales.

[39] Gunn: El Enfoque de Gunn Para el Tratamiento de la—Google Académico. https://scholar.google.com/scholar_lookup?title=The+Gunn+approach+to+the+treatment+of+chronic+pain&author=CC+Gunn&publication_year=1997&.

[40] Gunn C.C., Milbrandt W.E., Little A.S., Mason K.E. (1980). Dry needling of muscle motor points for chronic low-back pain: A randomized clinical trial with long-term follow-up. Spine.

[41] Chen J.T., Chung K.C., Hou C.R., Kuan T.S., Chen S.M., Hong C.Z. (2001). Inhibitory effect of dry needling on the spontaneous electrical activity recorded from myofascial trigger spots of rabbit skeletal muscle. Am. J. Phys. Med. Rehabil..

[42] Dommerholt J. (2011). Dry needling—Peripheral and central considerations. J. Man. Manip. Ther..

[43] Srbely J.Z., Dickey J.P., Lee D., Lowerison M. (2010). Dry needle stimulation of myofascial trigger points evokes segmental anti-nociceptive effects. J. Rehabil. Med..

[44] Hsieh J.-C., Tu C.-H., Chen F.-P., Chen M.-C., Yeh T.-C., Cheng H.-C., Wu Y.-T., Liu R.-S., Ho L.-T. (2001). Activation of the hypothalamus characterizes the acupuncture stimulation at the analgesic point in human: A positron emission tomography study. Neurosci. Lett..

[45] Biella G., Sotgiu M.L., Pellegata G., Paulesu E., Castiglioni I., Fazio F. (2001). Acupuncture produces central activations in pain regions. Neuroimage.

[46] Loizidis T., Nikodelis T., Bakas E., Kollias I. (2020). The effects of dry needling on pain relief and functional balance in patients with sub-chronic low back pain. J. Back Musculoskelet. Rehabil..

[47] Tsai C.T., Hsieh L.F., Kuan T.S., Kao M.J., Chou L.W., Hong C.Z. (2010). Remote effects of dry needling on the irritability of the myofascial trigger point in the upper trapezius muscle. Am. J. Phys. Med. Rehabil..

[48] Hsieh Y.L., Kao M.J., Kuan T.S., Chen S.M., Chen J.T., Hong C.Z. (2007). Dry needling to a key myofascial trigger point may reduce the irritability of satellite MTrPs. Am. J. Phys. Med. Rehabil..

[49] Simons D.G. (2004). Review of enigmatic MTrPs as a common cause of enigmatic musculoskeletal pain and dysfunction. J. Electromyogr. Kinesiol..

[50] Renan-Ordine R., Alburquerque-Sendín F., De Souza D.P.R., Cleland J.A., Fernández-De-Las-Penas C. (2011). Effectiveness of myofascial trigger point manual therapy combined with a self-stretching protocol for the management of plantar heel pain: A randomized controlled trial. J. Orthop. Sports Phys. Ther..

[51] Hou C.R., Tsai L.C., Cheng K.F., Chung K.C., Hong C.Z. (2002). Immediate effects of various physical therapeutic modalities on cervical myofascial pain and trigger-point sensitivity. Arch. Phys. Med. Rehabil..

[52] Acevedo González J.C., González A., Melzack J.R., Wall P. (2013). Ronald Melzack and Patrick Wall. La teoría de la compuerta: Más allá del concepto científico dos universos científicos dedicados al entendimiento del dolor. Rev. Soc. Española Dolor..

[53] Ziaeifar M., Arab A.M., Nourbakhsh M.R. (2016). Clinical Effectiveness of Dry Needling Immediately After Application on Myofascial Trigger Point in Upper Trapezius Muscle. J. Chiropr. Med..

[54] Rikards L. (2006). The effectiveness of non-invasive treatments for active myofascial trigger point pain: A systematic review of the literature. Int. J. Osteopath. Med..

[55] Gerber L.H., Shah J., Rosenberger W., Armstrong K., Turo D., Otto P., Heimur J., Thaker N., Sikdar S. (2015). Dry Needling Alters Trigger Points in the Upper Trapezius Muscle and Reduces Pain in Subjects With Chronic Myofascial Pain. PMR.

[56] Kietrys D.M., Palombaro K.M., Azzaretto E., Hubler R., Schaller B., Schlussel J.M., Tucker M. (2013). Effectiveness of dry needling for upper-quarter myofascial pain: A systematic review and meta-analysis. J. Orthop. Sports Phys. Ther..

[57] FISIOLOGIA ARTICULAR (6^a^ ED.) TOMO 2: MIEMBRO INFERIOR|I.A. KAPANDJI|Casa del Libro. https://www.casadellibro.com/libro-fisiologia-articular-6-ed-tomo-2-miembro-inferior/9788498354591/1965468?gclid=Cj0KCQjwl7qSBhD-ARIsACvV1X0ZzirQ6hz_6K7NK9OGTEWaQfPn2t59WqAo5aP1hbthNeT4w5n4jdQaAqciEALw_wcB.

[58] Nadler S.F., Malanga G.A., Bartoli L.A., Feinberg J.H., Prybicien M., Deprince M. (2002). Hip muscle imbalance and low back pain in athletes: Influence of core strengthening. Med. Sci. Sports Exerc..

[59] Declaración de Helsinki de la AMM—Principios éticos Para las Investigaciones Médicas en Seres Humanos—WMA—The World Medical Association. https://www.wma.net/es/policies-post/declaracion-de-helsinki-de-la-amm-principios-eticos-para-las-investigaciones-medicas-en-seres-humanos/.

[60] Pérez-Palomares S., Jiménez-Sánchez C., Serrano-Herrero I., Herrero P., Calvo S. (2021). Is Instrumental Compression Equally Effective and Comfortable for Physiotherapists and Physiotherapy Students than Manual Compression? A Comparative Cross-Sectional Study. Int. J. Environ. Res. Public Health.

[61] Martínez De La Iglesia J., Herrero R.D., Vilches M.C.O., Taberné C.A., Colomer C.A., Luque R.L. (2001). Spanish language adaptation and validation of the Pfeiffer’s questionnaire (SPMSQ) to detect cognitive deterioration in people over 65 years of age. Med. Clin..

[62] Mejuto-Vázquez M.J., Salom-Moreno J., Ortega-Santiago R., Truyols-Domínguez S., Fernández-de-Las-Peñas C. (2015). Cambios a corto plazo en el dolor de cuello, sensibilidad generalizada al dolor por presión y rango de movimiento cervical después de la aplicación de punción seca en el punto gatillo en pacientes con dolor de cuello mecánico agudo: Un ensayo clínico aleatorizado [la corrección publicada aparece en J Orthop Sports Phys Ther. 2015 Apr;45(4):329]. J. Orthop. Sports Phys. Ther..

[63] Seidel H. (1993). Selected health risks caused by long-term, whole-body vibration. Am. J. Ind. Med..

[64] Edwards J., Knowles N. (2003). Superficial dry needling and active stretching in the treatment of myofascial pain—A randomised controlled trial. Acupunct. Med..

[65] Boyles R., Fowler R., Ramsey D., Burrows E. (2015). Effectiveness of trigger point dry needling for multiple body regions: A systematic review. J. Man. Manip. Ther..

[66] Cagnie B., Castelein B., Pollie F., Steelant L., Verhoeyen H., Cools A. (2015). Evidence for the Use of Ischemic Compression and Dry Needling in the Management of Trigger Points of the Upper Trapezius in Patients with Neck Pain: A Systematic Review. Am. J. Phys. Med. Rehabil..

[67] Aguilera F.J.M., Martín D.P., Masanet R.A., Botella A.C., Soler L.B., Morell F.B. (2009). Immediate effect of ultrasound and ischemic compression techniques for the treatment of trapezius latent myofascial trigger points in healthy subjects: A randomized controlled study. J. Manip. Physiol. Ther..

[68] Roland M., Fairbank J. (2000). The Roland-Morris Disability Questionnaire and the Oswestry Disability Questionnaire. Spine.

[69] Badia X., Alonso J. (2007). La Medida de la Salud, Guia de Escalas de Medición en Español Edimac. http://ccuc.csuc.cat/record=b2870247~S23*cat.

[70] Bernardelli R.S., Santos B.C., Scharan K.O., Corrêa K.P., Silveira M.I.B., de Lima Moser A.D. (2021). Application of the refinements of ICF linking rules to the Visual Analogue Scale, Roland Morris questionnaire and SF-36. Cien. Saude Colet..

[71] Vanderweeën L., Oostendorp R.A.B., Vaes P., Duquet W. (1996). Pressure algometry in manual therapy. Man. Ther..

[72] Park G., Kim C.W., Park S.B., Kim M.J., Jang S.H. (2011). Reliability and Usefulness of the Pressure Pain Threshold Measurement in Patients with Myofascial Pain. Ann. Rehabil. Med..

[73] Tousignant M., Poulin L., Marchand S., Viau A., Place C. (2005). The Modified-Modified Schober Test for range of motion assessment of lumbar flexion in patients with low back pain: A study of criterion validity, intra- and inter-rater reliability and minimum metrically detectable change. Disabil. Rehabil..

[74] Irnich D., Behrens N., Gleditsch J.M., Stör W., Schreiber M.A., Schöps P., Vickers A., Beyer A. (2002). Immediate effects of dry needling and acupuncture at distant points in chronic neck pain: Results of a randomized, double-blind, sham-controlled crossover trial. Pain.

[75] Benito-De-Pedro M., Vallejo R.B.D.B., Losa-Iglesias M.E., Rodríguez-Sanz D., López-López D., López P.P., Mazoteras-Pardo V., Lobo C.C. (2020). Effectiveness of Deep Dry Needling vs. Ischemic Compression in the Latent Myofascial Trigger Points of the Shortened Triceps Surae from Triathletes on Ankle Dorsiflexion, Dynamic, and Static Plantar Pressure Distribution: A Clinical Trial. Pain Med..

[76] Espejo-Antúnez L., Tejeda J.F., Albornoz-Cabello M., Rodriguez-Mansilla J., de la Cruz-Torres B., Ribeiro F., Silva A.G. (2017). Punción seca en el tratamiento de los puntos gatillo miofasciales: Una revisión sistemática de ensayos controlados aleatorios. Complement. Ther. Med..

[77] Velázquez-Saornil J., Ruíz-Ruíz B., Rodríguez-Sanz D., Romero-Morales C., López-López D., Calvo-Lobo C. (2017). Efficacy of quadriceps vastus medialis dry needling in a rehabilitation protocol after surgical reconstruction of complete anterior cruciate ligament rupture. Medicine.

[78] Martín-Pintado-Zugasti A., Rodríguez-Fernández Á.L., Fernandez-Carnero J. (2016). Postneedling soreness after deep dry needling of a latent myofascial trigger point in the upper trapezius muscle: Characteristics, sex differences and associated factors. J. Back Musculoskelet. Rehabil..

[79] Kodama K., Takamoto K., Nishimaru H., Matsumoto J., Takamura Y., Sakai S., Ono T., Nishijo H. (2019). Analgesic Effects of Compression at Trigger Points Are Associated With Reduction of Frontal Polar Cortical Activity as Well as Functional Connectivity Between the Frontal Polar Area and Insula in Patients With Chronic Low Back Pain: A Randomized Trial. Front. Syst. Neurosci..

[80] Martín-Sacristán L., Calvo-Lobo C., Pecos-Martín D., Fernández-Carnero J., Alonso-Pérez J.L. (2022). Dry needling in active or latent trigger point in patients with neck pain: A randomized clinical trial. Sci. Rep..

[81] Benito-De-Pedro M., Becerro-de-Bengoa-Vallejo R., Losa-Iglesias M.E., Rodríguez-Sanz D., López-López D., Cosín-Matamoros J., Martínez-Jiménez E., Calvo-Lobo C. (2019). Effectiveness between Dry Needling and Ischemic Compression in the Triceps Surae Latent Myofascial Trigger Points of Triathletes on Pressure Pain Threshold and Thermography: A Single Blinded Randomized Clinical Trial. J. Clin. Med..

[82] Calvo-Lobo C., Pacheco-Da-Costa S., Hita-Herranz E. (2017). Efficacy of Deep Dry Needling on Latent Myofascial Trigger Points in Older Adults With Nonspecific Shoulder Pain: A Randomized, Controlled Clinical Trial Pilot Study. J. Geriatr. Phys. Ther..

[83] Llamas-Ramos R., Pecos-Martín D., Gallego-Izquierdo T., Llamas-Ramos I., Plaza-Manzano G., Ortega-Santiago R., Cleland J., Fernández-De-Las-Peñas C. (2014). Comparison of the short-term outcomes between trigger point dry needling and trigger point manual therapy for the management of chronic mechanical neck pain: A randomized clinical trial. J. Orthop. Sports Phys. Ther..

[84] Ganesh G.S., Singh H., Mushtaq S., Mohanty P., Pattnaik M. (2016). Effect of cervical mobilization and ischemic compression therapy on contralateral cervical side flexion and pressure pain threshold in latent upper trapezius trigger points. J. Bodyw. Mov. Ther..

[85] Pecos-Martin D., Ponce-Castro M.J., Jiménez-Rejano J.J., Nunez-Nagy S., Calvo-Lobo C., Gallego-Izquierdo T. (2019). Immediate effects of variable durations of pressure release technique on latent myofascial trigger points of the levator scapulae: A double-blinded randomised clinical trial. Acupunct. Med..

[86] Ziaeifar M., Arab A.M., Karimi N., Nourbakhsh M.R. (2014). The effect of dry needling on pain, pressure pain threshold and disability in patients with a myofascial trigger point in the upper trapezius muscle. J. Bodyw. Mov. Ther..

